# Anormalidades Cardíacas nas Síndromes Hipereosinofílicas

**DOI:** 10.36660/abc.20240190

**Published:** 2024-11-07

**Authors:** Viviane Tiemi Hotta, Rafael Ruas Nastari, Gardênia da Silva Lobo Oishi, Alexandre Eiji Kayano, Juliana Alzira Gonzales Oliveira, Ruiza Gonçalves Rocha, Ana Olga Mocumbi, Fernanda Salles Seguro, José Eduardo Krieger, Fábio Fernandes, Vera Maria Cury Salemi

**Affiliations:** 1 Hospital das Clínicas Faculdade de Medicina Universidade de São Paulo São Paulo SP Brasil Instituto do Coração do Hospital das Clínicas da Faculdade de Medicina da Universidade de São Paulo, São Paulo, SP – Brasil; 2 Fleury Medicina e Saúde São Paulo SP Brasil Fleury Medicina e Saúde, Grupo Fleury, São Paulo, SP – Brasil; 3 Instituto do Câncer do Estado de São Paulo Faculdade de Medicina Universidade de São Paulo São Paulo SP Brasil Instituto do Câncer do Estado de São Paulo – Hospital das Clínicas HCFMUSP, Faculdade de Medicina – Universidade de São Paulo, São Paulo,SP – Brasil; 4 Hospital Israelita Albert Einstein São Paulo SP Brasil Hospital Israelita Albert Einstein, São Paulo, SP – Brasil; 5 Instituto Nacional de Saúde Marracuene Moçambique Instituto Nacional de Saúde, Marracuene – Moçambique; 6 Universidade Eduardo Mondlane Maputo Moçambique Universidade Eduardo Mondlane, Maputo – Moçambique; 7 Hospital Sírio Libanês São Paulo SP Brasil Hospital Sírio Libanês, São Paulo, SP – Brasil

**Keywords:** Cardiomiopatia Restritiva, Eosinófilos, Insuficiência Cardíaca, Síndrome Hipereosinofílica

## Abstract

A Hipereosinofilia (HE) é definida como uma contagem de eosinófilos superior a 1500 células/microL no sangue periférico em dois exames, realizados com intervalo mínimo de um mês e/ou confirmação anatomopatológica de HE, com eosinófilos compreendendo mais de 20% de todas as células nucleadas da medula óssea. A Síndrome hipereosinofílica (SHE) indica a presença de HE com comprometimento de órgãos por ação eosinofílica, podendo ser classificada como primária (ou neoplásica), secundária (ou reativa) e idiopática. O comprometimento cardíaco ocorre em até 5% dos casos na fase aguda e em 20% na fase crônica da doença, variando de casos oligossintomáticos até miocardite aguda fulminante ou cardiomiopatia restritiva crônica (endomiocardite de Loeffler). No entanto, o grau de disfunção cardíaca não se correlaciona diretamente com o grau de eosinofilia. O envolvimento cardíaco na SHE ocorre em três fases: necrótica inicial, trombótica e necrótica final. Pode se manifestar como insuficiência cardíaca, arritmias e fenômenos tromboembólicos. O diagnóstico de cardiopatia é baseado em métodos de imagem multimodalidade, com ênfase na importância do ecocardiograma transtorácico (ETT). Em pacientes com janela acústica limitada, podem ser utilizados agentes de contraste ultrassonográfico, que permitem melhor visualização das bordas endocárdicas e da região ventricular apical. Técnicas para análise da deformação miocárdica podem evidenciar redução do strain em segmentos apicais e preservação nos demais segmentos (*reverse apical sparing*). A ressonância magnética cardíaca permite a caracterização do realce tardio subendocárdico de gadolínio, e a biópsia endomiocárdica é considerada o padrão ouro no diagnóstico de cardiopatia. O tratamento é baseado na etiologia da SHE.

## Graphical abstract

**Figura Central:**
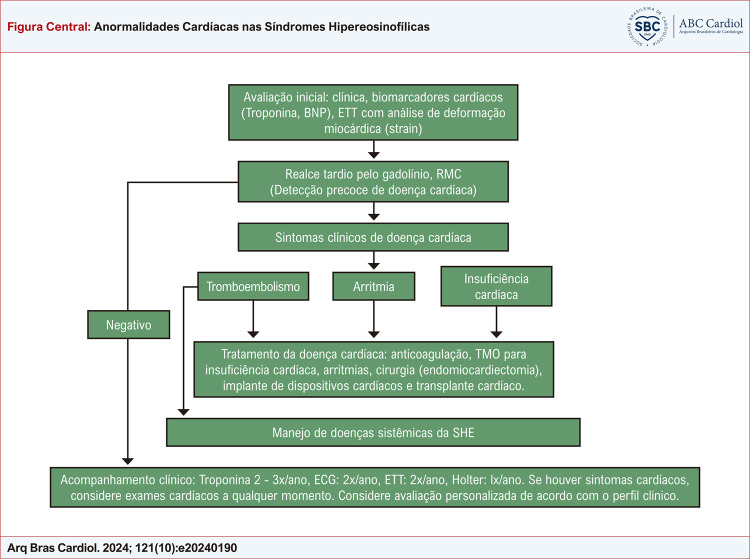
: Anormalidades Cardíacas nas Síndromes Hipereosinofílicas

Abordagem sugerida em pacientes com Síndromes Hipereosinofílicas (SHE) e suspeita de acometimento cardíaco. BNP: peptídeo natriurético tipo B; ETT: ecocardiograma transtorácico; RMC: ressonância magnética cardíaca; TMO: tratamento medicamentoso otimizado.

## Introdução

Os distúrbios eosinofílicos abrangem um grupo de condições com fisiopatologia altamente heterogênea, apresentações clínicas e prognósticos variáveis, que vão desde casos assintomáticos a graves e complexos.^1,2^ Nas últimas duas décadas, houve avanços na compreensão de mecanismos moleculares, refinamento de critérios diagnósticos, classificação e avaliação de opções terapêuticas. No entanto, ainda existem inúmeras lacunas e desafios na avaliação das síndromes hipereosinofílicas (SHE) na prática clínica. O prognóstico da doença depende da causa e do mecanismo da eosinofilia, da gravidade da disfunção orgânica, do diagnóstico preciso e da resposta terapêutica.^1,2^

Em 2012, o *International Cooperative Working Group on Eosinophil Disorders,*^1^ liderado pela *Medical University of Vienna*, propôs um novo consenso sobre a terminologia associada a essas condições. Seguindo essas recomendações, a hipereosinofilia (HE) foi definida como uma contagem de eosinófilos superior a 1500 células/microL no sangue periférico em dois exames, com intervalo de pelo menos um mês, e/ou confirmação anatomopatológica de HE, ou seja, eosinófilos constituem mais de 20% de todas as células nucleadas na medula óssea, com extensa infiltração de eosinófilos nos tecidos afetados ou deposição significativa de proteínas granulares de eosinófilos (cristais de Charcot Leyden), conforme avaliação de um patologista.^3,4^

A SHE é definida pela presença de HE e consequente comprometimento e/ou disfunção de órgãos devido à ação dos eosinófilos, na ausência de outras causas que justifiquem essas lesões.

Atualmente, existem várias classificações para caracterizar a SHE. Uma das mais aceitas descreve três formas: primária (ou neoplásica), caracterizada por um distúrbio clonal da linhagem mieloide; secundária (ou reativa), a forma mais comum, resultante de uma condição prévia, que incluem reações adversas a medicamentos, infecções ou neoplasias (Tabela 1);^5,6^ e idiopática, na qual a HE permanece sem uma causa atribuível após investigação completa. Em alguns casos, a HE não leva a comprometimentos orgânicos, sendo denominada HE de significado indeterminado ou simplesmente HE, que não requer tratamento; apenas acompanhamento clínico.

**Tabela 1:** – Causas secundárias mais comuns de síndromes hipereosinofílicas

Causas mais comuns		
Alergias	Atopia grave	Asma eosinofílica
		Dermatites atópicas
		Eczema
Infecciosa	Parasitas helmínticos	*Toxocara canis*
		*Ascaris lumbricoides*
		*Strongyloides stercoralis*
		Esquistossomose
	Ectoparasitas	Sarcoptes scabiei
	Fúngica	Aspergilose
		Coccidioidomicose
	Viral	HIV
Neoplásica	Hematológica	Linfoma de células T
		(angioimunoblástica, periférica)
		Leucemia Mieloide Crônica
		Linfoma de Hodgkin
	Tumores sólidos	Adenocarcinoma
		Tumores de cabeça e pescoço
**Causas menos comuns**		
Dermatológica	Não atópica	Síndrome de Wells
Autoimune/Vasculite		Vasculite por pênfigo bolhoso
		Granulomatose eosinofílica com poliangiite
		Doença inflamatória IgG4
		Poliarterite nodosa
		Lúpus eritematoso sistêmico
		Fascite eosinofílica
Imunodeficiências primárias		Síndrome de OMENN
		Síndrome de hiper IgE
Gastrointestinais		Doenças inflamatórias intestinais
		Pancreatite crônica
		Doença celíaca
Diversos		Embolia de colesterol
		Radiação

Fonte: Butt et al.^5^ e Curtis et al.^6^

Em relação ao envolvimento cardíaco, uma revisão realizada em 1975^4^ revelou que 95% dos indivíduos com SHE apresentavam sinais clínicos ou achados de autópsia indicativos de cardiopatia. No entanto, com os avanços no diagnóstico e tratamento precoce da síndrome, um estudo multicêntrico recente demonstrou uma redução do envolvimento cardíaco, tanto na fase aguda (até 5%) quanto na fase crônica, quando acomete 20% dessa população.^7^

A manifestação de doença cardíaca em pacientes com SHE pode ser idiossincrática, uma vez que não há correlação definida entre níveis elevados específicos de eosinófilos ou a duração da HE e o início de complicações cardíacas.^8,9^ O espectro da SHE varia amplamente, com estudos sugerindo que cerca de um terço dos pacientes com a forma primária são propensos a desenvolver fibrose endomiocárdica,^10^ enquanto aqueles diagnosticados com SHE reativa têm risco reduzido de envolvimento cardíaco.^11^

O presente artigo analisa as implicações cardíacas associadas à SHE, incluindo relatos de casos e artigos de revisão sobre a função dos eosinófilos, SHE e Endomiocardiofibrose (EMF).

### Fisiopatologia

Na hematopoiese humana, as células-tronco se diferenciam em progenitores mieloides e linfoides. Os progenitores mieloides se diferenciarão ainda mais em glóbulos vermelhos (eritrócitos), plaquetas, monócitos (que se tornarão macrófagos) e granulócitos (neutrófilos, eosinófilos e basófilos). Diversos fatores de crescimento e citocinas, como a eritropoietina (EPO) e o fator estimulador de colônias de granulócitos-macrófagos (GM-CSF), desempenham papéis cruciais na regulação da hematopoiese. A IL-5 é específica para diferenciação de granulócitos em eosinófilos e desempenha um papel fundamental no desenvolvimento desta linhagem celular.^12,13^

Os eosinófilos são vitais para combater infecções, auxiliando no reparo e reestruturação de tecidos. Além disso, desempenham um papel na vigilância de tumores e estabelecem interações sinérgicas com várias outras células especializadas do sistema imunológico para garantir a homeostase fisiológica.^14,15^ No geral, existem quatro mecanismos principais associados a um aumento de eosinófilos no sangue periférico ou infiltração de tecidos: proliferação clonal,^16^ proliferação policlonal, aumento da sobrevivência de eosinófilos ou migração alterada de eosinófilos.

O primeiro mecanismo resulta de defeitos moleculares em células-tronco hematopoiéticas ou defeitos na transmissão de sinais de receptores que orientam a eosinopoiese. Em certos casos, os eosinófilos são as principais células afetadas, como visto na leucemia eosinofílica crônica e em neoplasias mieloides/linfoides com eosinofilia e rearranjo do receptor de tirosina quinase, com este último representado principalmente pelo rearranjo PDGFRA. Em outras situações, os eosinófilos são apenas uma das linhagens celulares em expansão, conforme observado, por exemplo, na leucemia mieloide crônica. Na expansão policlonal, a eosinofilia é reativa ou secundária a um fator que induz a superprodução de IL-5, levando à diferenciação específica e exacerbada de granulócitos. Em raras ocasiões, a eosinofilia acentuada pode surgir de uma população clonal de linfócitos, conhecida como variante linfocítica da síndrome hipereosinofílica.^17,18^

Em relação à migração e sobrevivência, múltiplos processos envolvendo citocinas e moléculas de adesão (como integrinas) estão relacionados à progressão de eosinófilos da medula óssea para o sangue/tecidos. Alguns medicamentos que interferem nessas vias podem levar à eosinofilia.^19^ A elevação da IL-5 também é descrita como um dos mecanismos que aumentam a sobrevivência de eosinófilos *in vitro*.^20^

### Apresentação clínica e alterações cardíacas

Os eosinófilos ativados podem causar danos aos tecidos por meio da liberação de grânulos tóxicos e citocinas ou pelo recrutamento de células inflamatórias.^21^ A infiltração extensa de tecidos pode levar a danos e fibrose se a proliferação for significativa.^22^

A apresentação clínica varia de casos oligossintomáticos a miocardite necrosante eosinofílica aguda fulminante ou cardiomiopatia restritiva crônica (também conhecida como endomiocardite ou cardiomiopatia de Loeffler).^23,24^

O envolvimento cardíaco pode variar, dependendo do estágio do comprometimento miocárdico.^24^ Na fase inicial, há infiltração eosinofílica do subendocárdio, muitas vezes silenciosa. Durante esse estágio, é descrita a ocorrência de miocardite fulminante, com áreas de necrose extensa e insuficiência cardíaca (IC) rapidamente progressiva.^1^ Microêmbolos podem se formar na superfície endocárdica, e fenômenos tromboembólicos, além de hemorragias conjuntivais ou subungueais também podem ser identificadas nesse estágio.^25^

No estágio subsequente, ocorre a formação de trombo intracavitário,^1^ com potencial para acidentes vasculares e isquemia de membros.^4,26^ No terceiro e último estágio, a inflamação eosinofílica estabelecida leva à fibrose subendocárdica, principalmente na região trabecular e nos tratos de entrada, geralmente preservando o trato de saída. Essa fibrose difusa pode resultar em restrição miocárdica,^1^ manifestando-se com sintomas de dispneia e sinais de insuficiência cardíaca esquerda ou direita com fração de ejeção (FE) preservada.^26,27^ A restrição do folheto valvar, resultante da fibrose, leva à regurgitação das valvas átrioventriculares, sendo a valva mitral a mais acometida.^28^ A fibrose é irreversível e pode ser o estágio em que o paciente é diagnosticado pela primeira vez com SHE.^24^

A apresentação clínica se assemelha muito à EMF em termos de características clínicas, achados ecocardiográficos e de ressonância magnética cardíaca (RMC). Alguns autores acreditam que a EMF e a SHE representam apresentações clínicas dentro do espectro da mesma doença. No entanto, a SHE exibe manifestações sistêmicas e ocorre mais comumente em climas temperados, enquanto a EMF apresenta predominantemente manifestações cardíacas, ocorrendo em regiões tropicais e subtropicais.

Arritmias ventriculares podem ocorrer como resultado de fibrose no sistema de condução ou devido à fibrose miocárdica. Infartos do miocárdio são raros, mas podem apresentar-se como resultado de um fenômeno embólico de um trombo no ápice ventricular ou no trato de saída do ventrículo esquerdo.^29^

Os sintomas cardíacos geralmente evoluem ao longo de semanas a meses, mas podem se estender para além desse período.^30^ É essencial enfatizar que o desenvolvimento de doença cardíaca na SHE pode ser imprevisível, com estágios sobrepostos, e não existe uma relação clara entre a ocorrência e a gravidade do envolvimento cardíaco e sintomas gerais ou de outros órgãos.^29,31^

### Diagnóstico etiológico da hipereosinofilia

O diagnóstico etiológico da SHE deve ser guiado por dados obtidos do histórico médico e exame físico. Geralmente, na avaliação inicial, deve-se realizar a exclusão de eosinofilia reativa. Para isso, uma ferramenta útil é a dosagem da imunoglobulina E sérica, um importante mediador de eosinófilos. Em condições reativas, espera-se um aumento de seus níveis séricos.^30^

No contexto da EH reativa, destacam-se as reações medicamentosas (AINEs, aspirina, antibióticos etc.) e as condições alérgicas, responsáveis por 80% dos casos. Além disso, vale mencionar várias outras condições, como infecções, especialmente estrongiloidíase;^32,33^ doenças autoimunes, com foco na granulomatose eosinofílica com poliangiite; doenças neoplásicas; e outras condições mais raras, como ateroembolia de colesterol.^5^

Uma vez que as principais condições secundárias tenham sido descartadas, especialmente para pacientes com comprometimento significativo de órgãos, como comprometimento cardíaco, é fundamental colaborar com um hematologista para a investigação de condições clonais específicas desta especialidade.

Uma informação interessante e facilmente obtida é a medição dos níveis séricos de vitamina B12 e triptase, uma vez que níveis elevados apoiam a possibilidade de eosinofilia clonal.^34^

### Diagnóstico de alterações cardíacas

Com o desenvolvimento de novas tecnologias em imagem cardiovascular, principalmente a ecocardiografia e a RMC, a avaliação não invasiva se tornou uma alternativa interessante para o diagnóstico do acometimento cardíaco em pacientes com SHE.

### Achados laboratoriais

A avaliação laboratorial de pacientes com SHE inclui hemograma completo, níveis séricos de vitamina B12, esfregaço de sangue periférico, imunoglobulinas séricas, troponinas, CKMB e investigação laboratorial para infecções parasitárias.^2,3^

A medição de biomarcadores cardíacos, como troponinas e CKMB, pode fornecer informações precoces sobre lesão miocárdica, correlacionar-se com a ocorrência de desfechos e indicar pacientes que desenvolverão envolvimento cardíaco. Esses biomarcadores são válidos para a avaliação e monitoramento de pacientes com complicações cardíacas secundárias à SHE, incluindo na fase aguda, e podem ser usados no monitoramento da resposta terapêutica e na estimativa do prognóstico do paciente.^1,2^

### Eletrocardiograma

O eletrocardiograma (ECG) é uma ferramenta útil para a detecção de anormalidades cardíacas na SHE; no entanto, ele não revela alterações específicas.^2,3,8^ Estima-se que cerca de um terço dos pacientes com complicações cardíacas secundárias à SHE apresentam alterações eletrocardiográficas.^1,2^

Os achados eletrocardiográficos mais comuns incluem inversão da onda T, sinais de sobrecarga atrial esquerda, bloqueio atrioventricular de primeiro grau, bloqueio incompleto do ramo direito e sinais de sobrecarga ventricular esquerda.^2,3,8^ Outras anormalidades encontradas no ECG incluem extrassístoles ventriculares, episódios de taquicardia ventricular e arritmias supraventriculares, que podem estar presentes na fase aguda da doença.^8^

### Ecocardiograma transtorácico

O ETT é uma ferramenta importante para o diagnóstico e monitoramento de pacientes com complicações cardíacas secundárias à SHE, por se tratar de um método não invasivo de primeira linha, que permite a avaliação da anatomia cardíaca, além da função e hemodinâmica cardiovascular.^3,8^

**Figura 1:**
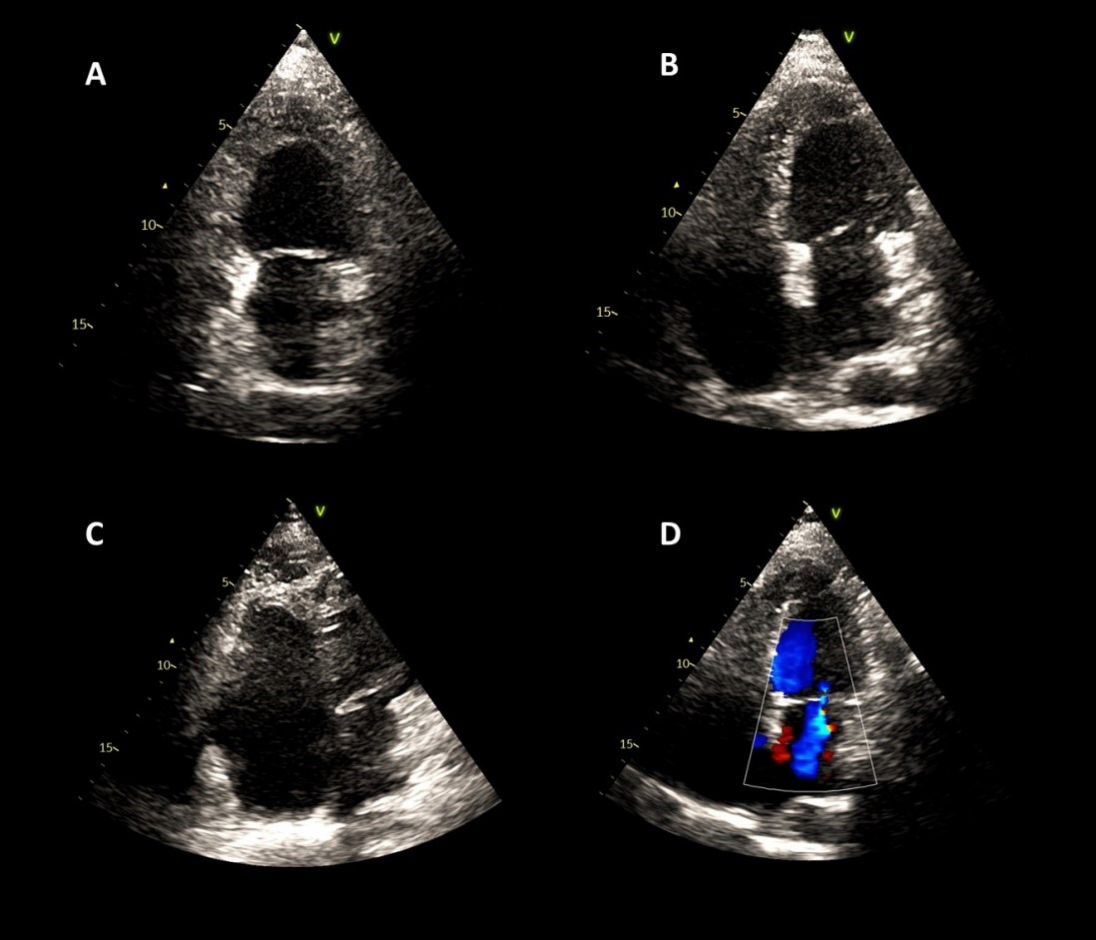
– Imagens de ecocardiograma transtorácico de um paciente diagnosticado com SHE e obliteração apical do ventrículo esquerdo no corte apical de 2 câmaras (A), corte apical de 4 câmaras (B) e do ventrículo direito no corte apical modificado para avaliação do VD (C). Observa-se insuficiência mitral de grau discreto ao Doppler colorido.

Durante a fase inicial do envolvimento cardíaco secundário à SHE (fase inflamatória/necrótica), o ecocardiograma pode não mostrar alterações óbvias ou pode exibir leve aumento da ecogenicidade na região subendocárdica.^1^ Nas fases trombótica e fibrótica da doença, podem ser observados achados ecocardiográficos típicos da SHE, como espessamento endomiocárdico, formação de trombo intracavitário, obliteração fibrótica de um ou ambos os ápices ventriculares, dilatação de um ou ambos os átrios, disfunção da valva atrioventricular devido ao envolvimento do aparelho subvalvar, regurgitação mitral secundária à restrição do movimento do folheto mitral posterior e disfunção diastólica com um padrão tipicamente restritivo (Figura 1).^1-3,8^ Alguns pacientes podem apresentar derrame pericárdico. O ETT pode ser aprimorado com agentes de ultrassom para melhor visualização de possíveis trombos apicais e diagnóstico diferencial com outras condições cardíacas, como cardiomiopatia hipertrófica apical e miocárdio não compactado.^1,2^

Técnicas que permitem a análise da deformação miocárdica, como ecocardiografia de *speckle tracking* (STE), oferecem informações sobre o comprometimento sistólico ventricular precoce em pacientes com FE ventricular esquerda preservada. O comprometimento apical predominante (*reverse apical sparing*) pode ser observado no mapa polar da deformação longitudinal do ventrículo esquerdo. Esse padrão também é comum na cardiomiopatia hipertrófica, que apresenta deformação apical reduzida, mas diferentemente da SHE, o valor global é preservado.^35^

Além disso, pacientes com SHE podem desenvolver quadro clínico compatível com insuficiência cardíaca com FE preservada e cardiomiopatia restritiva. Nesses casos, a ecocardiografia também permite a análise da função diastólica, de pressões de enchimento do ventrículo esquerdo e de pressões da artéria pulmonar.^24^

A ecocardiografia tridimensional (Eco 3D), por sua vez, apresenta melhor correlação com a RMC para avaliação das dimensões das câmaras cardíacas e FE do que o eco bidimensional, além de permitir melhor avaliação da região apical. A análise dos volumes atriais e ventriculares é realizada a partir da aquisição de um bloco volumétrico que permite melhor alinhamento dos planos sagital, coronal e transverso, possibilitando que a região apical seja totalmente incluída nos cálculos volumétricos. Além de apresentar melhor correlação com os resultados obtidos pela RMC, o Eco 3D permite melhor varredura da região apical, em busca por trombos ventriculares, que podem estar presentes em pacientes com miocardiopatias restritivas, endomiocardiofibrose e insuficiência cardíaca com fração de ejeção reduzida.^35-37^

### Ressonância magnética cardíaca

A RMC permite análise cardíaca morfológica e funcional de alta qualidade com excelente resolução espacial. Além disso, promove avaliação tecidual pela técnica de realce tardio pelo gadolínio, que evidencia sinais de necrose, inflamação e fibrose, além da quantificação de volumes extra e intracelulares, relevantes em doenças miocárdicas restritivas e infiltrativas. A quantificação da fibrose miocárdica está diretamente relacionada à remodelação ventricular, à ocorrência de arritmias e a um prognóstico piorado em diferentes condições cardíacas.

**Figura 2:**
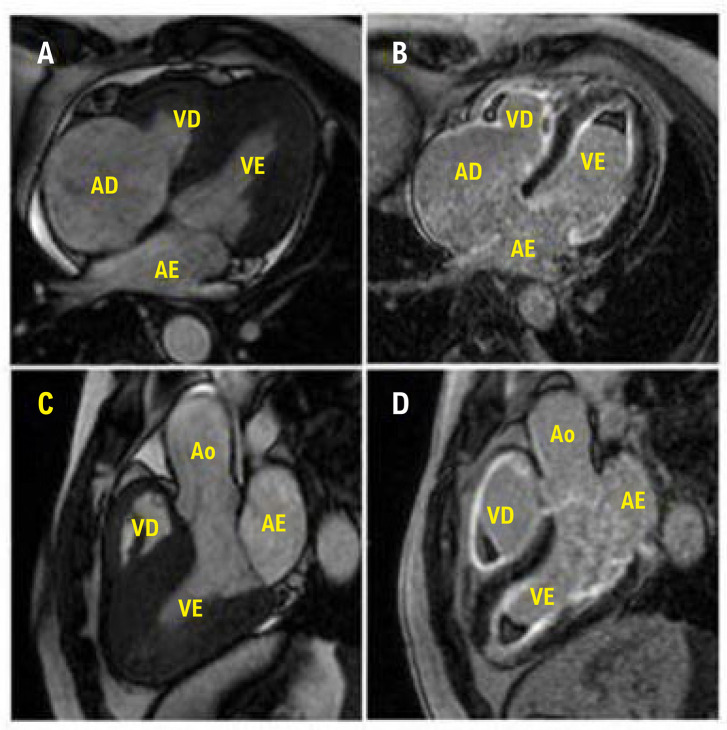
– Imagem de ressonância magnética cardíaca de um paciente com SHE e anormalidades cardíacas. Eixo longitudinal quatro câmaras com deposição de tecido fibroso endocárdico apical biventricular e microtrombos no átrio direito (A); Imagens para avaliação de fibrose pela técnica de realce tardio pelo gadolínio (RTG) (B). Eixo longitudinal da via de saída do ventrículo esquerdo com deposição de tecido fibroso endocárdico apical biventricular (C) ce avaliado pela técnica de RTG (D). Ao: aorta; AE: átrio esquerdo; VE: ventrículo esquerdo; AD: átrio direito; VD: ventrículo direito.

Na SHE, a RMC permite a detecção de realce tardio subendocárdico de gadolínio nos estágios iniciais da doença, mesmo na ausência de sintomas cardiovasculares e alterações ecocardiográficas.^1,3^ Em estágios avançados, o realce pode apresentar um padrão transmural (Figura 2).^1,7^

Além disso, a RMC também permite a detecção de trombos apicais intracavitários em pacientes com SHE e avaliação precisa da espessura pericárdica, importante na rara associação de SHE e pericardite constritiva.^1,35^

### Tomografia computadorizada cardíaca

A tomografia computadorizada cardíaca representa uma opção diagnóstica para pacientes com contraindicações para RMC, sendo uma ferramenta útil para a identificação de trombos intracavitários.^1,3^

### Biópsia endomiocárdica

A biópsia endomiocárdica permanece sendo o padrão ouro para o diagnóstico do envolvimento cardíaco secundário à SHE. Ela revela infiltrados de eosinófilos ou grânulos eosinofílicos no miocárdio, além de fibrose e trombos murais.^1,3,4^ No entanto, a relação risco-benefício para o paciente deve ser cuidadosamente considerada devido à possibilidade de complicações e efeitos iatrogênicos.^2,4^

### Diagnóstico diferencial

As manifestações cardíacas na Síndrome Hipereosinofílica (SHE) assemelham-se aos achados em pacientes com EMF. Enquanto alguns autores acreditam que essas condições se enquadram no espectro clínico da mesma doença, a SHE e a EMF diferem em aspectos epidemiológicos e clínicos, conforme descrito na Tabela 2.^38,39^ A cardiomiopatia hipertrófica apical também deve ser considerada no diagnóstico diferencial quando o ventrículo esquerdo é afetado em pacientes com envolvimento cardíaco em SHE semelhante a EMF.

**Tabela 2:** – Diagnóstico diferencial de pacientes com SHE e Endomiocardiofibrose

	SHE	EMF
Epidemiologia	Desconhecido	Mais da metade dos casos são relatados em países da África Subsaariana,^1^^-^^5^ e menos comumente no Sul da Ásia e América Latina.
Exposição Ambiental	HE reativa, especialmente reações a medicamentos (AINEs, aspirina, antibióticos etc.) e condições alérgicas. Outras condições como infecções, principalmente estrongiloidíase (NCCN, 2021).	Pode estar associada à pobreza, desnutrição, dieta, fatores ambientais e infecções em um indivíduo suscetível para dar origem a um processo inflamatório que leva a danos endomiocárdicos e formação de cicatrizes.^6^
Genética	Desconhecido	Um estudo de dois centros foi projetado para investigar a variação no sistema HLA. Os tipos de HLA classe I (HLA-A, -B, -C) e classe II (DRB1, DQB1) foram determinados em 71 pacientes com EMF grave e 137 controles de Uganda e Moçambique. Comparados aos controles etnicamente pareados, os pacientes com EMF foram mais propensos do que os controles a ter o alelo HLA-B*58 em Moçambique (p-0,03) e o HLA-A*02:02 em Uganda (p = 0,005).^40^^,^^41^

Fonte: Grimaldi et al.^39^ SHE: Síndrome hipereosinofílica; EMF: endomiocardiofibrose.

### Conceitos gerais de tratamento

O tratamento deve ser direcionado à etiologia da SHE. Pacientes com envolvimento cardíaco, associado a alta morbidade e mortalidade, requerem terapia urgente.

A recomendação inicial é o uso de corticosteroides intravenosos em altas doses, seguido de manutenção oral com redução gradual ao longo de 2 a 3 meses. A redução gradual é aconselhada quando há controle sintomático e redução nos níveis de eosinófilos, com o valor alvo sendo incerto na literatura. A maioria dos autores sugere níveis de eosinófilos < 1500/L.^2^ No entanto, definir a etiologia subjacente é crucial, algumas etiologias como HE mieloide/SHE (neoplasia mieloide eosinofílica, incluindo aquelas associadas a rearranjos recorrentes) podem não responder bem a esta terapia e devem ser tratadas com terapia direcionada.^42^

Rearranjos envolvendo PDGFRA e PDGFRB, representantes primários de neoplasias mieloides/linfoides com eosinofilia e rearranjo do receptor de tirosina quinase, devem ser tratados com mesilato de imatinibe devido à sua maior eficácia (resposta hematológica geral em cerca de 85% dos casos e resposta completa em cerca de 64%).^3^ Parece haver uma resposta com baixas doses de mesilato de imatinibe, mas dada a raridade da condição, não há consenso na literatura sobre a dosagem ideal. Em geral, a maioria dos autores concorda em usar 100 a 400 mg/dia, resultando potencialmente em uma regressão completa do envolvimento cardíaco.^2^ Nesses pacientes, os corticosteroides devem ser usados no início do tratamento junto com o mesilato de imatinibe para prevenir o desenvolvimento de insuficiência cardíaca aguda devido à miocardite necrosante.^3^ Atualmente, não há um protocolo de descontinuação para o inibidor da tirosina quinase nessa população.

Outras opções podem ser usadas para várias etiologias, especialmente em combinação com corticosteroides (agentes preservadores de corticosteroides) ou administrados sequencialmente em pacientes mais resistentes a corticosteroides. Essas opções incluem hidroxiureia, ciclofosfamida, interferon-alfa, ciclosporina, metotrexato, alemtuzumabe e mepolizumabe.

Em casos mais refratários ou com alto risco de progredir para leucemia aguda (como aqueles envolvendo o rearranjo do FGFR1), o transplante alogênico de células-tronco pode ser necessário.^1^

À medida que nossa compreensão da fisiopatologia progride, há uma melhora correspondente nas ferramentas terapêuticas, como evidenciado pelo sucesso de terapias direcionadas, como o mesilato de imatinibe em PDGFRA e PDGFRB. Diversos outros alvos moleculares, como o anti-IL5 (*Mepolizumab*), anti-CD52 (*Alemtuzumab*) e inibidor de FGFR (*Pemigatinib*), ainda estão sob investigação para essa população.

### Tratamento das alterações cardíacas

Em relação ao tratamento cardíaco, não há ensaios clínicos randomizados para esse grupo de pacientes. Como a cardiomiopatia hipereosinofílica pode se manifestar em vários espectros, o tratamento deve ser direcionado à manifestação cardíaca apresentada, seguindo diretrizes nacionais e internacionais.

- Insuficiência cardíaca: a síndrome clínica de insuficiência cardíaca com FE preservada devido à obliteração apical, que leva a uma fisiopatologia restritiva, é a manifestação cardíaca mais frequente da SHE,^3^ e deve ser tratada de acordo com recomendações específicas para insuficiência cardíaca.^4^ Na fase aguda da doença, altas doses de corticoides podem ser utilizadas para a obtenção de melhores resultados. No entanto, essa intervenção deve ser realizada precocemente.^1^
- Choque cardiogênico: alguns relatos de caso na literatura mostram pacientes que necessitam de suporte circulatório mecânico ou bomba de balão intra-aórtico, combinados com agentes inotrópicos.^7-10^ Há um relato de caso de urgência de transplante cardíaco em um paciente de 38 anos com rápida progressão de miocardite eosinofílica necrosante.^11^
- Complicações tromboembólicas: eventos tromboembólicos ocorrem em até 25% dos pacientes com SHE.^2^ A anticoagulação com antagonista da vitamina K é indicada em pacientes com trombo intracavitário confirmado e/ou eventos tromboembólicos recorrentes, bem como pacientes após cirurgia cardíaca valvar.^1^ Na ausência de contraindicações clínicas, os anticoagulantes orais são indicados na presença de fibrilação atrial como profilaxia primária. Nesses pacientes, a aplicação do escore CHADS-VASc não é indicada. Embora o uso de anticoagulantes orais diretos tenha sido sugerido como alternativa, não há evidências documentadas de seu benefício para cardiomiopatia eosinofílica.^12^
- Doenças valvulares: devido à falta de evidências neste grupo de pacientes, as indicações para intervenção cirúrgica valvar devem seguir as diretrizes para o tratamento de patologias valvares em geral. A substituição da válvula mitral é o procedimento mais comumente realizado nestes pacientes,^1^ embora o reparo da válvula seja viável e frequentemente preferido nesta população, pois a decisão sobre qual a prótese de preferência não é clara.^3^ As próteses mecânicas apresentam maior risco de trombose obstrutiva nestes pacientes devido à intensa atividade pró-coagulante das células eosinofílicas.^2^ No entanto, mesmo em casos de bioprótese, a anticoagulação com varfarina é indicada, pois os pacientes com SHE têm maior associação com a formação de trombos e diminuição da longevidade da prótese biológica devido ao aumento da atividade inflamatória da doença subjacente. Por esse motivo, mesmo em pacientes submetidos à intervenção valvar, é importante monitorar os níveis de eosinofilia.^3^ A taxa de mortalidade em pacientes submetidos à intervenção valvar enquanto mantinham terapia medicamentosa dentro de 3 anos foi de 4%, em comparação com a mortalidade de 77% no grupo que não recebeu terapia medicamentosa após a cirurgia valvar.^2^
- Arritmias e morte cardíaca súbita: devido à quantidade significativa de substratos arritmogênicos em pacientes com SHE, mesmo sem o desenvolvimento de fibrose per se, eles podem desenvolver arritmias supraventriculares, arritmias ventriculares complexas e morte cardíaca súbita. Portanto, a consideração da colocação de cardioversor-desfibrilador implantável (CDI) deve ser feita, mesmo em casos de prevenção primária, considerando o controle da doença, a fração de ejeção e a extensão da fibrose.^1^
- Cardiomiopatia restritiva avançada: nesses pacientes, a ressecção cirúrgica do endocárdio fibrótico (ressecção endomiocárdica) pode ser considerada benéfica em comparação à terapia medicamentosa padrão, potencialmente melhorando a função diastólica e reduzindo os sintomas.^1^
- Pericardite constritiva: embora raro na literatura, o tratamento necessário para esses pacientes é a pericardiectomia.^2^

### Abordagem sugerida em pacientes com SHE e suspeita de envolvimento cardíaco

Devido ao potencial envolvimento cardíaco, todos os pacientes com SHE (primária ou secundária) devem ser submetidos a avaliação clínica para sinais e sintomas de insuficiência cardíaca, arritmias e eventos tromboembólicos. Além do histórico médico, os pacientes devem ser submetidos a medidas de biomarcadores cardíacos, como BNP e troponinas, bem como ECG e ETT (Figura Central). Quando a janela acústica do paciente é limitada, especialmente na região apical, o ETT com agentes de ultrassom aprimorados pode ser usado para melhor visualização das bordas endocárdicas e identificação do padrão de obliteração apical. Quando disponível, a análise da deformação miocárdica pela técnica STE é interessante, pois pode destacar um padrão de preservação apical reversa com preservação dos segmentos basal e médio (*reverse apical sparing*).

A RMC deve ser realizada em todos os pacientes com SHE e suspeita de envolvimento cardíaco devido à sua maior precisão diagnóstica e à possibilidade de detecção precoce de alterações cardíacas. A biópsia endomiocárdica pode ser considerada em pacientes com alta suspeita clínica de envolvimento cardíaco que apresentem dados inconclusivos de métodos de imagem cardíaca.

Todos os pacientes devem ser submetidos a tratamento sistêmico para SHE em conjunto com a equipe de hematologia. A abordagem terapêutica da síndrome de insuficiência cardíaca, seja com fração de ejeção preservada ou reduzida, arritmias e lesões valvares, deve seguir diretrizes específicas. A anticoagulação oral é indicada para pacientes com trombos intracardíacos, eventos tromboembólicos ou fibrilação atrial. O escore CHADS-VAsc não se aplica a esse grupo de pacientes. Apesar da escassez de estudos randomizados extensos para esse grupo de pacientes, novos anticoagulantes orais podem ser utilizados.

O tratamento cirúrgico para ressecção de fibrose apical, remoção de trombos e insuficiência de valvas atrioventriculares pode ser considerado para pacientes em classe funcional III ou IV da New York Heart Association, refratários ao tratamento clínico.

Em relação ao acompanhamento clínico, os pacientes com SHE devem ser submetidos a exames de imagem cardíaca (ecocardiografia e RMC) a qualquer momento na presença de sinais e sintomas de insuficiência cardíaca. Em pacientes assintomáticos, biomarcadores cardíacos e ECG devem ser avaliados a cada quatro a seis meses, e ecocardiografia e monitoramento Holter a cada seis a doze meses.

## Conclusões e perspectivas futuras

A SHE representa um grupo heterogêneo e complexo de doenças que podem envolver lesão miocárdica por meio de eosinófilos ativados liberando grânulos tóxicos e citocinas ou recrutando células inflamatórias. Alguns pacientes podem apresentar uma síndrome clínica restritiva semelhante àquelas com EMF (Fluxograma 1). Atualmente, o foco principal da pesquisa em condições de SHE é obter uma classificação mais clara e prática dos diferentes tipos de doenças eosinofílicas, identificação de novos biomarcadores e tratamentos mais eficazes. Estudos adicionais sobre o papel da SHE nessas síndromes, juntamente com o aumento do conhecimento sobre potenciais alterações cardíacas no contexto da SHE e o refinamento de métodos diagnósticos não invasivos, incluindo biomarcadores como BNP, NT-pro BNP, troponinas, galectina-3, interleucinas, ETT com estudo Doppler e análise da mecânica cardíaca, bem como RMC, podem fornecer novos insights sobre a fisiopatologia desse grupo de doenças.
