# Supplementary material for: Anormalidades Cardíacas nas Síndromes Hipereosinofílicas
Source: Arq Bras Cardiol. 2024 Nov 7;121(10):e20240190. [Article in Portuguese] doi: 10.36660/abc.20240190 (PMC11634209; doi:10.36660/abc.20240190)
Supplement: Translation

# Translation: Cardiac Abnormalities in Hypereosinophilic Syndromes

**DOI:** 10.36660/abc.20240190i

**Authors:** Viviane Tiemi Hotta, Rafael Ruas Nastari, Gardênia da Silva Lobo Oishi, Alexandre Eiji Kayano, Juliana Alzira Gonzales Oliveira Leguizamon, Ruiza Gonçalves Rocha, Ana Olga Mocumbi, Fernanda Salles Seguro, José Eduardo Krieger, Fábio Fernandes, Vera Maria Cury Salemi

**Affiliations:** 1 Brazil Instituto do Coração do Hospital das Clínicas da Faculdade de Medicina da Universidade de São Paulo, São Paulo, SP – Brazil; 2 Brazil Fleury Medicina e Saúde, Grupo Fleury, São Paulo, SP – Brazil; 3 Brazil Instituto do Câncer do Estado de São Paulo – Hospital das Clínicas HCFMUSP, Faculdade de Medicina – Universidade de São Paulo, São Paulo, SP –Brazil; 4 Brazil Hospital Israelita Albert Einstein, São Paulo, SP – Brazil; 5 Mozambique Instituto Nacional de Saúde, Marracuene – Mozambique; 6 Mozambique Universidade Eduardo Mondlane, Maputo – Mozambique; 7 Brazil Hospital Sírio Libanês, São Paulo, SP – Brazil

**Keywords:** Restrictive Cardiomyopathy, Eosinophils, Heart Failure, Hypereosinophilic Syndrome

## Abstract

Hypereosinophilia (HE) is defined as an eosinophil count exceeding 1500 cells/microL in peripheral blood in two tests, performed with an interval of at least one month and/or anatomopathological confirmation of HE, with eosinophils comprising more than 20% of all nucleated cells in the bone marrow. Hypereosinophilic syndrome (HES) indicates the presence of HE with organ involvement due to eosinophil action, which can be classified as primary (or neoplastic), secondary (or reactive), and idiopathic. Cardiac involvement occurs in up to 5% of cases in the acute phase and 20% of the chronic phase of the disease, ranging from oligosymptomatic cases to fulminant acute myocarditis or chronic restrictive cardiomyopathy (Loeffler endomyocarditis). However, the degree of cardiac dysfunction does not directly correlate with the degree of eosinophilia. The cardiac involvement of HES occurs in three phases: initial necrotic, thrombotic, and finally necrotic. It can manifest as heart failure, arrhythmias, and thromboembolic phenomena. The diagnosis of cardiopathy is based on multimodality imaging, with an emphasis on the importance of echocardiography (echo) as the primary examination. TTE with enhanced ultrasound agents can be used for better visualization, allowing greater accuracy in assessing ventricular apex, and myocardial deformation indices, such as longitudinal strain, may be reduced, especially in the ventricular apex (reverse apical sparing). Cardiac magnetic resonance imaging allows the characterization of subendocardial late gadolinium enhancement, and endomyocardial biopsy is considered the gold standard in diagnosing cardiopathy. Treatment is based on the etiology of HES.

## Introduction

Eosinophilic disorders encompass a group of conditions with highly heterogeneous pathophysiology, clinical presentations, and variable prognoses, ranging from asymptomatic cases to more severe and complex outcomes.^1,2^ Over the past two decades, there have been consistent advances in understanding molecular mechanisms, refining diagnostic criteria, classifying disorders, and evaluating therapeutic options. However, there still exist numerous gaps and challenges in the assessment of hypereosinophilic syndromes (HES) in clinical practice. The prognosis of the disease fundamentally depends on the cause and mechanism of eosinophilia, the severity of organ dysfunction, accurate diagnosis, and therapeutic response.^1,2^

In 2012, the International Cooperative Working Group on Eosinophil Disorders,^1^ led by the Medical University of Vienna, proposed a new consensus on terminology associated with these conditions. Following these recommendations, hypereosinophilia (HE) was defined as an eosinophil count exceeding 1500 cells/microL in peripheral blood in two tests with an interval of at least one month and/or anatomopathological confirmation of HE, meaning eosinophils constitute more than 20% of all nucleated cells in the bone marrow, extensive infiltration of eosinophils in affected tissues, or significant deposition of eosinophil granule proteins (Charcot Leyden crystals), as assessed by a pathologist.^3,4^

The HES is defined by the presence of HE and consequent impairment and/or dysfunction of organs due to eosinophil action, in the absence of other causes justifying these injuries.

Currently, there are several classifications to characterize HES. One of the most accepted describes three forms: primary (or neoplastic), characterized by a clonal disorder of the myeloid lineage; secondary (or reactive), the most common form, resulting from a previous condition such as adverse drug reactions, infections, or neoplasms (Table 1);^5,6^ and, idiopathic, where HE remains without an attributable cause after thorough investigation. In addition to the mentioned HES, other associated forms have also been described, making this topic even more complex. In certain cases, HE does not lead to organic impairments, being termed indeterminate significance HE or simply HE, which requires no treatment; only clinical follow-up is needed.

**Table 1:** – Most common secondary causes of hypereosinophilic syndromes

Most Common Causes		
Allergies	Major atopy	Eosinophilic asthma
Atopic dermatitis
Eczema
Infectious	Helminth parasites	*Toxocara canis*
		*Ascaris lumbricoides*
		*Strongyloides stercoralis*
		Schistosomiasis
	Ectoparasites	Sarcoptes scabiei
	Fungal	Aspergillosis
		Coccidioidomycosis
	Viral	HIV
Neoplasic	Hematological	T cell lymphoma
		(angioimmunoblastic, peripheral)
		Chronic Myeloid Leukemia
		Hodgkin lymphoma
	Solid Tumors	Adenocarcinoma
		Head and neck tumors
**Less Common Causes**		
Dermatological	Non-atopic	Wells´ syndrome
Autoimmune / Vasculitis		Bullous pemphigus vasculitis
		Eosinophilic granulomatosis with polyangiitis
		IgG4 Inflammatory disease
		Polyarteritis nodosa
		Systemic lupus erythematosus
		Eosinophilic Fasciitis
Primary Immunodeficiencies		OMENN syndrome
		Hyper IgE syndrome
Gastrointestinal		Inflammatory bowel diseases
		Chronic pancreatitis
		Celiac disease
Miscellaneous		Cholesterol embolism
		Radiation

Source: Butt et al.^5^ and Curtis et al.^6^

Regarding cardiac involvement, a review conducted in 1975^4^ revealed that 95% of individuals with HES showed clinical signs or autopsy findings indicative of cardiopathy. However, with advances in early diagnosis and treatment of the syndrome, a recent multicenter study demonstrated a reduction in cardiac involvement, both in the acute phase (up to 5%) and the chronic phase, when it affects 20% of this population.^7^

The manifestation of cardiac disease in patients with HES can be idiosyncratic, as there is no defined correlation between specific elevated levels of eosinophils or the duration of HE and the onset of cardiac complications.^8,9^ The spectrum of HES varies widely: studies suggest that about one-third of patients with the primary form are prone to developing endomyocardial fibrosis (EMF),^10^ while those diagnosed with reactive HES have mitigated the risk of cardiac involvement.^11^

This article reviews the cardiac implications associated with HES, including case reports and review articles concerning eosinophils’ function, HES and EMF.

### Pathophysiology

In human hematopoiesis, stem cells differentiate into myeloid and lymphoid progenitors. Myeloid progenitors will further differentiate into red blood cells (erythrocytes), platelets, monocytes (which will become macrophages), and granulocytes (neutrophils, eosinophils, and basophils). Various growth factors and cytokines, such as erythropoietin (EPO) and granulocyte-macrophage colony-stimulating factor (GM-CSF), play crucial roles in regulating hematopoiesis. IL-5 is specific for eosinophil differentiation and plays a fundamental role in the development of this cell lineage.^12,13^

Eosinophils are vital for combating infections and assisting in tissue repair and restructuring. Additionally, they play a role in tumor surveillance and establish synergistic interactions with various other specialized cells of the immune system to ensure physiological homeostasis.^14,15^ Overall, there are four main mechanisms associated with an increase in eosinophils in peripheral blood or tissue infiltration: clonal proliferation,^16^ polyclonal proliferation, increased eosinophil survival, or altered eosinophil migration.

The first mechanism results from molecular defects in hematopoietic stem cells or defects in signal transmission from receptors that guide eosinopoiesis. In certain cases, eosinophils are the primary affected cells, as seen in chronic eosinophilic leukemia and in myeloid/lymphoid neoplasms with eosinophilia and rearrangement of the tyrosine kinase receptor, with the latter represented mainly by PDGFRA rearrangement. In other situations, eosinophils are just one of the expanding cell lineages, as seen, for example, in chronic myeloid leukemia. In polyclonal expansion, eosinophilia is reactive or secondary to a factor that induces the overproduction of IL-5, leading to specific and exaggerated differentiation in granulocytes. In rare occasions, marked eosinophilia can arise from a clonal population of lymphocytes, known as the lymphocytic variant of hypereosinophilic syndrome.^17,18^

Regarding migration and survival, multiple processes involving cytokines and adhesion molecules (such as integrins) are related to the progression of eosinophils from the bone marrow to the blood/tissues. Some medications that interfere with these pathways can lead to eosinophilia.^19^ The elevation of IL-5 is also described as one of the mechanisms increasing the survival of eosinophils in vitro.^20^

### Clinical presentation and cardiac alterations

Activated eosinophils can cause tissue damage through the release of toxic granules and cytokines or by recruiting inflammatory cells.^21^ Extensive tissue infiltration can lead to damage and fibrosis if proliferation is significant.^22^

The clinical presentation varies from oligosymptomatic cases to fulminant acute eosinophilic necrotizing myocarditis or chronic restrictive cardiomyopathy (also known as endomyocarditis or Loeffler’s cardiomyopathy).^23,24^

Cardiac involvement can vary depending on the stage of myocardial damage.^24^ In the initial phase, there is eosinophilic infiltration of the subendocardium, often silent. However, some patients may evolve with fulminat myocarditis, with areas of extensive necrosis and quickly progressive heart failure (HF).^1^ Microemboli may form on the endocardial surface, and conjunctival or subungual hemorrhages may also be identified at this stage.^25^

In the subsequent stage, intracavitary thrombus formation occurs,^1^with the potential for strokes and limb ischemia.^4,26^ In the third and final stage, established eosinophilic inflammation leads to subendocardial fibrosis, primarily in the trabecular region and inflow tracts, usually sparing the outflow tract. This diffuse fibrosis can result in myocardial restriction,^1^ manifesting with dyspnea symptoms and signs of left or right heart failure with preserved ejection fraction (EF).^26,27^ Valve leaflet restriction due to fibrosis leads to valvular regurgitation, with mitral valve being most commonly affected.^28^ The fibrosis is irreversible and may be the stage at which the patient is first diagnosed with HES.^24^

The clinical presentation closely resembles EMF in terms of clinical features, echocardiographic and cardiac magnetic resonance imaging (CMRI) findings. Some authors believe that EMF and HES represent clinical presentations within the spectrum of the same disease. However, HES exhibits systemic manifestations and occurs more commonly in temperate climates, while EMF predominantly presents with cardiac manifestations and occurs in tropical and subtropical regions.

Ventricular arrhythmias can occur as a result of fibrosis in the conduction system or due to myocardial fibrosis. Myocardial infarctions are rare but can occur as a result of an embolic phenomenon from a thrombus at the ventricular apex or the left ventricular outflow tract.^29^

Cardiac symptoms typically evolve over weeks to months but can extend beyond that timeframe.^30^ It is essential to emphasize that the development of cardiac disease in HES can be unpredictable, with stages overlapping, and there is no clear relationship between the occurrence and severity of cardiac disease and other organs involvement.^29,31^

### Etiological diagnosis of hypereosinophilia

The etiological diagnosis of HES should be guided by data obtained from the medical history and physical examination. Generally, in the initial evaluation, the exclusion of reactive eosinophilia should be performed. For this purpose, a useful tool is the measurement of serum immunoglobulin E, an important mediator of eosinophils. In reactive conditions, an increase in serum levels is expected.^30^

Within the context of reactive HE, drug reactions (NSAIDs, aspirin, antibiotics, etc.) and allergic conditions stand out, accounting for 80% of all cases. It is also important to mention various other conditions, such as infections, especially strongyloidiasis;^32,33^ autoimmune diseases, with a focus on eosinophilic granulomatosis with polyangiitis; neoplastic diseases; and other rarer conditions, such as cholesterol atheroembolism.^5^

Once major secondary conditions have been ruled out, especially for patients with significant organ involvement such as cardiac impairment, it is crucial to collaborate with a hematologist for the investigation of specific clonal conditions in this specialty.

An interesting and easily obtainable information is the measurement of serum levels of vitamin B12 and tryptase, as elevated levels support the possibility of clonal eosinophilia.^34^

### Diagnosis of cardiac alterations

With the development of new technologies in cardiovascular imaging, primarily echocardiography and CMRI, non-invasive assessment has become an interesting alternative for evaluating these patients.

### Laboratory findings

The laboratory evaluation of patients with HES includes a complete blood count, serum vitamin B12 levels, peripheral blood smear, serum immunoglobulins, troponins, CKMB, and laboratory investigation for parasitic infections.^2,3^

The measurement of cardiac biomarkers such as troponins and CKMB can provide early information about myocardial injury, correlate with the occurrence of outcomes, and indicate patients who will develop cardiac involvement. These biomarkers are valid for the assessment and monitoring of patients with cardiac complications secondary to HES, including in the acute phase. They can be used in monitoring therapeutic response and estimating patient prognosis.^1,2^

### Electrocardiogram

The electrocardiogram (ECG) is a useful tool for detecting cardiac abnormalities in HES; however, it does not reveal specific changes.^2,3,8^ It is estimated that about one-third of patients with cardiac complications secondary to HES exhibit electrocardiographic alterations.^1,2^

The most common electrocardiographic findings include T-wave inversion, signs of left atrial overload, first-degree atrioventricular block, incomplete right bundle branch block, and signs of left ventricular overload.^2,3,8^ Other abnormalities found on the ECG include ventricular extrasystoles, episodes of ventricular tachycardia, and supraventricular arrhythmias, which may be present in the acute phase of the disease.^8^

### Transthoracic echocardiogram

Transthoracic echocardiogram (TTE) is an important tool for the diagnosis and monitoring of patients with cardiac complications secondary to HES, as it is a first-line non-invasive method that allows for the evaluation of cardiac anatomy, function and cardiovascular hemodynamics.^3,8^

During the initial phase of cardiac involvement secondary to HES (inflammatory/necrotic phase), the echocardiogram may show no obvious changes or may exhibit mild increased echogenicity in the subendocardial region.^1^ In the thrombotic and fibrotic phases of the disease, typical echocardiographic findings of HES can be observed, such as endomyocardial thickening, intracavitary thrombus formation, fibrotic obliteration of one or both ventricular apices, dilation of one or both atria, atrioventricular valve dysfunction due to subvalvular apparatus involvement, mitral regurgitation secondary to restricted motion of the posterior mitral leaflet, and diastolic dysfunction with a typically restrictive pattern (Figure 1).^1-3,8^ Some patients may present pericardial effusion. TTE can be enhanced with ultrasound agents for better visualization of possible apical thrombi and differential diagnosis with other heart conditions such as apical hypertrophic cardiomyopathy and non-compacted myocardium.^1,2^

**Figure 1:** – Transthoracic echocardiogram images of a patient diagnosed with HES and apical obliteration of the left ventricle in the apical 2-chamber view (A), apical 4-chamber view (B) and from the right ventricle to the apical 4-chamber view directed towards the RV (C). A mild degree of mitral regurgitation is observed on color Doppler echocardiography.

Techniques that allow the analysis of myocardial deformation, such as strain using speckle tracking echocardiography (STE), provide information about early ventricular systolic impairment in patients with preserved left ventricular EF. Predominant apical impairment (reverse apical sparing) can be observed in the polar map of left ventricular longitudinal strain. This pattern is also common in hypertrophic cardiomyopathy, which shows reduced apical strain, but unlike HES, the global value is preserved.^35^

Moreover, patients with HES may develop a clinical picture compatible with heart failure with preserved EF and restrictive cardiomyopathy. In these cases, echocardiography also allows the analysis of diastolic function, left ventricular filling pressures, and pulmonary artery pressures.^24^

Three-dimensional echocardiography (3D Echo), in turn, has a better correlation with CMR for the evaluation of cardiac chamber dimensions and EF than two-dimensional echo, in addition to allowing a better assessment of the apical region. The analysis of atrial and ventricular volumes is performed from the acquisition of a Full Volume dataset that allows better alignment of sagittal, coronal, and transverse planes, enabling the apical region to be fully included in volumetric calculations. In addition to showing a better correlation with results obtained by CMR, 3D Echo allows a better scan of the apical region in the search for ventricular thrombi, which may be present in patients with restrictive cardiomyopathies, endomyocardial fibrosis, and heart failure with reduced ejection fraction.^35-37^

### Cardiac magnetic resonance imaging

CMRI allows high-quality morphological and functional cardiac analysis with excellent spatial resolution. It also promotes tissue evaluation using the late gadolinium enhancement technique, which identifies signs of necrosis, inflammation, and fibrosis and also the quantification of extra- and intracellular volumes, which are relevant in restrictive and infiltrative myocardial diseases. The quantification of myocardial fibrosis is directly related to ventricular remodeling, the occurrence of arrhythmias, and worse prognosis in different heart conditions.

In HES, CMRI enables the detection of subendocardial late gadolinium enhancement in the early stages of the disease, even in the absence of cardiovascular symptoms and echocardiographic changes.^1,3^ In advanced stages, enhancement may present a transmural pattern (Figure 2).^1,7^

**Figure 2:** – Cardiac magnetic resonance imaging of a patient with HES and cardiac abnormalities. Four chamber view with biventricular apical endocardial fibrous tissue deposition and microthrombi in the right atrium (A) with late gadolinium enhancement (LGE) CMRI (B). Left ventricular outflow tract view with biventricular apical endocardial fibrous tissue deposition (C) with LGE (D). Ao,:aorta; LA: left atrium; LV: left ventricle; RA: right atrium; RV: right ventricle.

Furthermore, CMRI also allows the detection of intracavitary apical thrombi in patients with HES and precise evaluation of pericardial thickness, which is important in the rare association of HES and constrictive pericarditis.^1,35^

### Cardiac computed tomography

Cardiac computed tomography represents a diagnostic option for patients with contraindications for CMR and is a useful tool for identifying intracavitary thrombi.^1,3^

### Endomyocardial biopsy

Endomyocardial biopsy remains the gold standard method for diagnosing cardiac involvement secondary to HES. It reveals eosinophil infiltrates or eosinophilic granules in the myocardium, in addition to fibrosis and mural thrombi.^1,3,4^ However, the risk-benefit relationship with the patient must be carefully considered due to the possibility of complications and iatrogenic effects.^2,4^

### Differential diagnosis

Cardiac manifestations in Hypereosinophilic Syndrome (HES) resemble findings in patients with EMF. While some authors believe that these conditions fall within the clinical spectrum of the same disease, HES and EMF differ in epidemiological and clinical aspects, as described in Table 2.^38,39^ Apical hypertrophic cardiomyopathy also must be considered in the differential diagnosis when the left ventricle is affected in patients with cardiac involvement in HES resembling EMF.

**Table 2:** – Differential diagnosis of patients with HES and endomyocardial fibrosis

	HES	EMF
Epidemiology	Unknown	More than half of the cases are reported in sub-Saharan African countries^1^^-^^5^ and less commonly in South Asia and Latin America.
Environmental Exposure	Reactive HE, especially drug reactions (NSAIDs, aspirin, antibiotics, etc.) and allergic conditions. Other conditions such as infections, mainly strongyloidiasis (NCCN, 2021).	It may be associated with poverty, malnutrition, diet, environmental factors, and infections in a susceptible individual to give rise to an inflammatory process that leads to endomyocardial damage and scar formation.^6^
Genetics	Unknown	A two-center study was designed to investigate variation in the HLA system HLA class I (HLA-A, -B, -C) and class II (DRB1, DQB1) types were determined in 71 patients with severe EMF and 137 controls from Uganda and Mozambique. Compared to ethnically matched controls, EMF patients were more likely than controls to have the HLA-B*58 allele in Mozambique (p-0.03) and the HLA-A*02:02 in Uganda (p = 0.005).^40^^,^^41^

Source: Grimaldi et al.^39^ HES: Hypereosinophilic Syndrome; EMF: endomyocardial fibrosis.

### General concepts of treatment

The treatment should be targeted toward the etiology of HES. Patients with cardiac involvement, associated with high morbidity and mortality, require urgent therapy.

The initial recommendation is the use of high-dose intravenous corticosteroids, followed by oral maintenance with tapering over 2 to 3 months. Tapering is advised when there is symptomatic control and a reduction in eosinophil levels, with the target value being uncertain in the literature. Most authors suggest eosinophil levels < 1500/L.^2^ However, defining the underlying etiology is crucial; some etiologies as myeloid HE/ HES (eosinophilic myeloid neoplasm, including those associated with recurrent rearrangements), may not respond well to this therapy and should be treated with targeted therapy.^42^

Rearrangements involving PDGFRA and PDGFRB, primary representatives of myeloid/lymphoid neoplasms with eosinophilia and rearrangement of the tyrosine kinase receptor, should be treated with imatinib mesylate due to its higher efficacy (overall hematologic response in about 85% of cases, and complete response in about 64%).^3^ There seems to be a response with low doses of imatinib mesylate, but given the rarity of the condition, there is no consensus in the literature regarding the optimal dosage. In general, most authors agree on using 100 to 400 mg/day, potentially resulting in a complete regression of cardiac involvement.^2^ In these patients, corticosteroids should be used at the beginning of treatment along with imatinib mesylate to prevent the development of acute heart failure due to necrotizing myocarditis.^3^ There is currently no discontinuation protocol for the tyrosine kinase inhibitor in this population.

Other options can be used for various etiologies, especially in combination with corticosteroids (corticosteroid-sparing agents) or administered sequentially in patients more resistant to corticosteroids. These options include hydroxyurea, cyclophosphamide, interferon-alpha, cyclosporine, methotrexate, alemtuzumab, and mepolizumab.

In cases that are more refractory or have a high risk of progressing to acute leukemia (such as those involving the FGFR1 rearrangement), allogeneic stem cell transplantation may be required.^1^

As our understanding of the pathophysiology progresses, there is a corresponding improvement in therapeutic tools, as evidenced by the success of targeted therapies such as imatinib mesylate in PDGFRA and PDGFRB. Numerous other molecular targets, such as the anti-IL5 (Mepolizumab), anti-CD52 (Alemtuzumab), and FGFR inhibitor (Pemigatinib), are still under investigation for this population.

### Treatment of cardiac alterations

Regarding cardiac treatment, there are no randomized controlled trials for this group of patients. Since hypereosinophilic cardiomyopathy can manifest in various spectrums, treatment should be directed towards the presented cardiac manifestation, following national and international guidelines.

• Heart Failure: The clinical syndrome of heart failure with preserved EF due to apical obliteration leading to restrictive pathophysiology is the most frequent cardiac manifestation in HES,^3^ and it should be treated according to specific recommendations for heart failure.^4^ In the acute phase of the disease, high doses of corticosteroids can be used for better results. However, this intervention should be performed early.^1^

• Cardiogenic Shock: There are reported cases in the literature where patients require mechanical circulatory support or intra-aortic balloon pump combined with inotropic agents.^7-10^ There is a case report of heart transplantation urgency in a 38-year-old patient rapidly progressing with necrotizing eosinophilic myocarditis.^11^

• Thromboembolic Complications: Thromboembolic events occur in up to 25% of HES patients.^2^ Vitamin K antagonist anticoagulation is indicated in patients with confirmed intracavitary thrombus and/or recurrent thromboembolic events, as well as in patients after valvular cardiac surgery.^1^ In the absence of clinical contraindications, oral anticoagulants are indicated in the presence of atrial fibrillation as primary prophylaxis. In these patients, the application of the CHADS-VASc score is not indicated. Although the use of direct oral anticoagulants has been suggested as an alternative, there is no documented evidence of their benefit for eosinophilic cardiomyopathy.^12^

• Valvular Diseases: Due to the lack of evidence in this patient group, indications for valvular surgical intervention should follow guidelines for the treatment of valvular pathologies in general. Mitral valve replacement is the most commonly performed procedure in these patients,^1^ although valve repair is feasible and often preferred in this population, as the decision on the preferred prosthesis is unclear.^3^ Mechanical prostheses pose a higher risk of obstructive thrombosis in these patients due to the intense procoagulant activity of eosinophilic cells.^2^ However, even in cases of bioprosthesis, warfarin anticoagulation is indicated since HES patients have a higher association with thrombus formation and decreased biological prosthesis longevity due to increased inflammatory activity of the underlying disease. For this reason, even in patients undergoing valve intervention, it is important to monitor eosinophilia levels.^3^ The mortality rate in patients undergoing valve intervention while maintaining clinical treatment within 3 years was 4%, compared to 77% mortality in the group not receiving medical treatment after valve surgery.^2^

• Arrhythmias and sudden cardiac death: Due to the significant amount of arrhythmogenic substrates in patients with HES, even without the development of fibrosis per se, they may develop supraventricular arrhythmias, complex ventricular arrhythmias and sudden cardiac death. Therefore, the consideration of implantable cardioverter-defibrillator (ICD) placement should be made, even in cases of primary prevention, considering disease control, ejection fraction, and extent of fibrosis.^1^

• Advanced restrictive cardiomyopathy: In these patients, surgical resection of the fibrotic endocardium (endomyocardial resection) may be considered beneficial compared to standard drug therapy, potentially improving diastolic function and reducing symptoms.^1^

• Constrictive pericarditis: Although rare in the literature, the required treatment for these patients is pericardiectomy.^2^

### Suggested approach in patients with HES and suspected cardiac involvement

Due to the potential cardiac involvement, all patients with HES (primary or secondary) should undergo clinical evaluation for signs and symptoms of heart failure, arrhythmias, and thromboembolic events. In addition to medical history, patients should undergo cardiac biomarker measurements such as BNP and troponins, as well as ECG and TTE (Central Illustration). When the patient’s acoustic window is limited, especially in the apical region, TTE with enhanced ultrasound agents can be used for better visualization of endocardial borders and identification of the apical obliteration pattern. When available, the analysis of myocardial deformation by the STE technique is interesting, as it can highlight a reverse apical sparing pattern with preservation of basal and mid segments (reverse *apical sparing*).

CMRI should be performed on all patients with HES and suspected cardiac involvement due to its higher diagnostic accuracy and the possibility of early detection of cardiac alterations. Endomyocardial biopsy may be considered in patients with a high clinical suspicion of cardiac involvement who present inconclusive data from cardiac imaging methods.

All patients should undergo systemic treatment for HES in conjunction with the hematology team. The treatment of cardiac complications should be performed according to the predominant involvement. The treatment of heart failure syndrome, whether with preserved or reduced ejection fraction, arrhythmias, and valvular lesions, should follow specific guidelines. Oral anticoagulation is indicated for patients with intracardiac thrombi, thromboembolic events, or atrial fibrillation. The CHADS-VAsc score does not apply to this group of patients. Despite the lack of extensive randomized studies for this patient group, new oral anticoagulants may be used.

Surgical treatment for the resection of apical fibrosis, removal of thrombi, and insufficiency of atrioventricular valves may be considered for patients in New York Heart Association functional class III or IV, refractory to clinical treatment.

Regarding clinical follow-up, patients with HES should undergo cardiac imaging (echocardiography and CMRI) at any time in the presence of signs and symptoms of heart failure. In asymptomatic patients, cardiac biomarkers and ECG should be measured every four to six months, and echocardiography and Holter monitoring every six to twelve.

## Conclusions and future perspectives

HES represents a heterogeneous and complex group of diseases that may involve myocardial injury through activated eosinophils releasing toxic granules and cytokines or recruiting inflammatory cells. Some patients may present with a restrictive clinical syndrome similar to those with EMF (Flowchart 1). Currently, the main focus of research in HES conditions is to achieve a clearer and more practical classification and characterization of different types of eosinophilic diseases, the identification of new biomarkers, and more effective treatments. Further studies on the role of HES in these syndromes, along with increased knowledge about potential cardiac alterations in the context of HES and refinement of non-invasive diagnostic methods, including biomarkers such as BNP, NT-pro BNP, troponins, galectin-3, interleukins, TTE with Doppler study, and cardiac mechanics analysis, as well as CMRI may provide new insights into the pathophysiology of this group of diseases.
